# Misrepresenting the “MAP” Literature Does Little to Advance Child Abuse Prevention: A Critical Commentary and Response to Farmer, Salter, and Woodlock

**DOI:** 10.1177/15248380251332197

**Published:** 2025-04-11

**Authors:** Craig A. Harper, Rebecca Lievesley, Nadine McKillop, Stephanie Price, Rachel Murphy, Ellie Woodward, Harriet Dymond, Christian Götzl

**Affiliations:** 1 Nottingham Trent University, UK; 2 University of the Sunshine Coast, Sippy Downs, QLD, Australia; 3 University of Central Lancashire, England, UK; 4 Safer Living Foundation, Nottingham, UK; 5 University of Ulm, Germany

**Keywords:** attractions to children, minor attraction, prevention, sexuality

## Abstract

In a recent paper published in *Trauma, Violence, & Abuse*, Farmer et al. purport to have critically examined the academic use of the term “Minor Attracted Persons” (MAPs) in published social science research. In the paper, their stated aims are to “understand how this body of scholarship defines and uses the terminology of MAP, conceptualizes sexual interest in minors, and its relationship with child sex offending, and the implications for child protection and safeguarding” (p. 4079). However, we believe that there are significant problems in their handling of this topic, their depth of analysis, and the selective reporting of research in the review that may demonstrate an inherent confirmation bias.

In a recent paper published in *Trauma, Violence, & Abuse*, Farmer et al. (2024) purport to have critically examined the academic use of the term “Minor Attracted Persons” (MAPs) in published social science research. In the paper, their stated aims are to “understand how this body of scholarship defines and uses the terminology of MAP, conceptualizes sexual interest in minors, and its relationship with child sex offending, and the implications for child protection and safeguarding” (p. 4079). However, we believe that there are significant problems in their handling of this topic, their depth of analysis, and the selective reporting of research in the review that may demonstrate an inherent confirmation bias.

Our concerns about Farmer et al.’s (2024) paper are twofold. First, there is selective reporting and omission of key “MAP” literature that paints an incomplete picture of this body of work, while centering non-empirical blogs and news reports in support of their broader argument. Second, those involved in “MAP” research are systematically misquoted, selected extracts from papers are presented out of context, and key findings are misinterpreted in a way that suggests a fundamental misunderstanding and misrepresentation of this body of work. We turn our attention to each of these issues below.

## Selective Reporting

Throughout Farmer et al.’s review, there is evidence of the selective reporting and interpretation of key areas of the “MAP” literature (particularly in relation to why “MAP” is used as a label in this work), and the partial quotation of authors engaged in “MAP” research.

### Why Do Researchers Use the Term “MAP”?

A central aspect of the argument advanced by Farmer et al. is that the use of the “MAP” label represents a concerted effort among researchers (with implied support or encouragement from organizations led by “MAPs” themselves) to destigmatize sexual attractions to children and “normalize” pedophilia. Although this use of the label is common across the literature (see, e.g., Walker & Panfil, 2017), it is important to stress that any stigma-related use of “MAP” is limited to discussions about attraction patterns rather than sexual behavior. Stigma reduction is also not the primary driver of the use of the “MAP” label for many authors. Indeed, in critiquing the destigmatization argument, Farmer et al. say:

It is also self-evident from the explosive public backlash whenever the MAPs term is used in policy or public debate that the term is not less stigmatized than “pedophile.” It is therefore curious why these scholars have embraced the term since their primary rationale for its use is not supported. (p. 4085)

There are several issues with this appraisal, though. First, the use of “MAP” within the academic literature pre-dates the spark for the “backlash” described by Farmer et al. (namely, the public outcry over an interview given by Allyn Walker to the Prostasia Foundation, 2021). Second, the assumed “primary rationale for its use” (stigma reduction) is in actuality tied to the central concern of Farmer et al.—that of sexual abuse prevention. That is, in using a term that is not tied to diagnostic criteria (or the conflations to offending that are inherent to these in social discourses; see, e.g., Feelgood & Hoyer, 2008), it is easier to discuss the difference between attraction and action, and to talk to potential prevention service users in a manner that gives them a sense of agency and control over the behavioral manifestations of their sexual interests in the service of harm reduction. Third, Farmer et al. appear to assume that “MAP” is no less stigmatized than diagnostic alternatives. Although this may appear to be the case if considering the fervor of discussions related to the “MAP” label on social media platforms, no academic work has been conducted to compare the levels of stigma associated with these different labels. It is, therefore, important to not mistake the reaction of some online communities for the opinions of society at large, in light of a lack of comparative data.

Given that Farmer et al. were explicit in their search for definitions of “MAP” in their review, it is noteworthy that one key definition used in multiple papers (which are cited in Farmer et al.’s review) is largely ignored.^1^ Those using this definition use “MAP” in their work to simply identify anybody who has an attraction to children, with “MAP” being used as a broad label owing to the oft-reported non-exclusivity of attractions to specific age categories (e.g., Martijn et al., 2020). This use does not relate to the supposed goals of some “MAP” scholars for destigmatization, and its consideration may: (a) lead to a less contentious or hyperbolic conclusion about the use of “MAP” as a descriptive label for people with attractions to children and (b) significantly reduce the level of confusion as to why some researchers have used the label in academic papers. In perhaps the most explicitly stated version of this use, Lievesley and Lapworth (2022) state:

The phrase “minor attraction” acts as an umbrella term to describe a range of chronophilic orientations. A chronophilia is a distinct type of sexual attraction pattern that varies as a function of the ages of preferred sexual targets (Seto, 2017). The most studied chronophilic category is pedophilia, which is defined as a primary or exclusive sexual attraction to pre-pubertal children, typically between the ages of 3 and 10 years (Blanchard et al., 2009). However, Seto’s (2017) model of chronophilias takes a much broader view and acknowledges that some people may have sexual preferences for younger infants (nepiophilia), pubescent children aged 11–14 years (hebephilia), or older minors who, depending on the legal code of a given jurisdiction, may be below the age of consent (ephebophilia). [. . .] For the purposes of this paper, we consider “minor attraction” to encompass the nepiophilic, pedophilic, and hebephilic attraction categories. (p. 880)

Notably, this definition is absent, despite the fact that Lievesley and Lapworth (2022) are actually cited by Farmer et al. in the same section where they review the various definitions of the “MAP” label, and thus, the omission of a fuller presentation of this definition is a significant oversight that skews perceptions about how the term “MAP” is used. Similar non-stigma-related broader definitions are also explicitly provided in a range of other peer-reviewed publications (see Garant and Proulx, 2024; Lievesley & Harper, 2022; Lievesley et al., 2023; McKillop & Price, 2023; Schmidt & Niehaus, 2022; Sorrentino & Abramowitz, 2021), with several of these missing from Farmer et al.’s review.

### Partial Quotation

There is evidence throughout Farmer et al.’s review of partial quotation that is, we believe, designed to portray arguments being stated by “MAP” researchers without their proper context. For example, Lievesley et al. (2023) are cited as stating:

. . . the MAPs in our sample may have over-prioritized mental health and stigma-related treatment targets and downplayed the extent to which they require support with managing their sexual attractions.

This quotation is presented by Farmer et al. to cast doubt on the truthfulness and accuracy of mental health-related concerns in the original authors’ work. However, in *the very next sentence*, Lievesley et al. (2023, p. 512) add:

However, our findings are concordant with prior unpublished informal surveys of the MAP community (e.g., B4U-ACT, 2011), theoretical accounts of appropriate MAP treatment approaches (Lievesley & Harper, 2022), qualitative analyses of barriers to MAPs seeking help (Dymond & Duff, 2020; Goodier & Lievesley, 2018; Grady et al., 2019; Levenson & Grady, 2019), and research into MAP wellbeing (Elchuk et al., 2022; Jahnke et al., 2015; Lievesley et al., 2020).

With this proper context, a consistent pattern of findings emerges from multiple research teams using different social scientific methods. This pattern highlights that mental health concerns are an important treatment target for “MAP” participant samples. This selective reporting and partial quotation from Farmer et al., thus, might be characterized as an attempt to mislead readers about the scale of the evidence for mental health-related concerns within this community and to maximize doubt in favor of their risk-related argument. This is not the only place where Farmer et al. provide partial quotes. They cite Walker and Panfil (2017) as arguing that “. . . primary attraction to minors does not appear to have a clear causal relationship to committing person offenses against children, in that minor attraction by itself seems neither necessary nor sufficient to explain interpersonal forms of sexual offending,” without identifying how this is contextualized within a discussion of the multifaceted causes of child sexual abuse, and the long-observed characteristics of those who cause sexual harm. For example, it is well known that the sexual abuse of children often involves a combination of factors that both motivate (e.g., sexual attractions to children or unmet sexual needs) and facilitate offending behavior (e.g., pro-criminal attitudes and offense-supportive beliefs about sexual activity with children; see Seto, 2019).

### Missing Literature

We appreciate Farmer et al. stating their inclusion criteria for the rapid review (peer-reviewed journal papers published in English between 2019 and 2023, and which use “MAP” terminology in titles and/or abstracts). However, this approach prevents a complete picture of the “MAP” literature from being presented. We know that some reviewers and editors share Farmer et al.’s discomfort with the “MAP” terminology and request this be removed from such prominent places despite it featuring throughout the rest of the paper. Acknowledging that we were aware of papers not included in the review, we conducted a rudimentary Google Scholar search using the following terms: “minor attracted person” OR “minor-attracted person” OR “minor attracted people” OR “minor-attracted people.” We list papers that were omitted by Farmer et al. in Table 1.

**Table 1.** Sources That Were Omitted From Farmer et al.’s Review, but Which Use “MAP” Terminology.

Citation	Title	Key findings
Cohen et al. (2018)	Comparison of self-identified minor-attracted persons who have and have not successfully refrained from sexual activity with children	“MAPs” who sexually offend are more likely to exhibit antisocial traits and more positive attitudes about adult–child sex, poor mental health, and attractions to boys.
Johnston (2017)	The unintended effect of mandatory reporting laws and an increased risk to a protected population	A lack of clarity around mandatory reporting can scupper treatment engagement efforts with “MAPs” seeking preventative support in the community.
Knack et al. (2019)	Primary and secondary prevention of child sexual abuse	This is a review paper identifying the benefits of prevention services in the community and barriers to treatment access.
Levenson et al. (2019)	Preventing sexual abuse: Perspectives of minor-attracted persons about seeking help	Characteristics of helpful therapeutic encounters include clinician non-judgment, improved clinician knowledge, and viewing clients in a person-centered and holistic way.
Sorrentino and Abramowitz (2021)	Minor-attracted persons: A neglected population	This is a review paper concluding that the development of a stronger understanding of attractions to children can decrease stigma, promote wellness, and prevent abuse.
Harper and Lievesley (2022)	Exploring the ownership of child-like sex dolls	“MAPs” who own sex dolls are less sexually preoccupied than non-owner “MAPs.” Inconsistent differences exist between the groups on self-reported offending proclivity.
Lievesley et al. (2022)	Primary health professionals’ beliefs, experiences, and willingness to treat minor-attracted persons	Primary health professionals are more likely than mental health professionals to view “MAPs” as dangerous, unable to control behaviors, and that sexual attractions are an avoidable choice.
Schmidt and Niehaus (2022)	Outpatient therapists’ perspectives on working with persons who are sexually interested in minors	Stigmatizing attitudes and a perceived lack of specific competence were negatively related to therapists’ willingness to treat “MAPs.”
Appel (2023)	Unconventional harm reduction interventions for minor-attracted persons	This is a review of possible risk-reduction implications for sex robots, haptic devices, and synthetic child pornography not involving real children.
Jahnke et al. (2024) ^a^	Secret-keeping in therapy by clients who are sexually attracted to children	Therapist reactions—not disclosure in isolation—impact “MAP” experiences of therapy.
Amelung et al. (2024) ^a^	The viewing reaction time as a diagnostic tool for pedohebephilia in the dunkelfeld	Viewing time can act as a non-intrusive diagnostic tool for the identification of pedohebephilic attractions within the community.

a This paper has a 2024 publication date but was published online within Farmer et al.’s rapid review timeframe.

### Confirmation Bias, Circular Citation, and Ideological Conflicts of Interest

Throughout our reading of the review, we detected confirmation bias in the arguments being presented. This is evident in: (a) the consistency between the interpretative framing of Farmer et al.’s review and the prior public positions taken by at least one of the review’s authors, (b) the reliance on popular press sources that are heavily cited throughout their article (e.g., UnHerd, VICE, Salon, The Post Millennial, Fox News, and The Daily Mail), and (c) the provision of an incomplete and misrepresentative view of this area of scholarship. As just one demonstration of this point, Farmer et al. state:

However, discussions among “MAPs” began to expand to include advocacy for the legalization of cartoon child sexual abuse material, access to child sexual abuse dolls, and, in some cases, the normalization of contact offending (Salter & Hanson, 2021). During this same period, internet safety agencies began reporting significant trading of child sexual abuse material on Twitter (Thalen, 2020). (p. 4081)

The references included within this section include self-citations to one of the authors of Farmer et al.’s review that does not actually cite any data pertaining to the use of child-like sex dolls (indeed, the word “dolls” does not actually appear at any point of the cited chapter). Furthermore, the Thalen (2020) citation refers to an online article published by The Daily Dot (a small online media organization) that is almost exclusively written about one of the review author’s personal tweets about Twitter’s rules on discussions of pedophilia. These citations, therefore, represent a form of unreliable circular referencing that, while appearing to provide credence to the point being made, offer no external validation to the broader argument. In so doing, the authors also omit arguments made from the “MAP” literature that run counter to their argument but which are directly linked to thinking more imaginatively about preventing the sexual abuse of children (for “MAP” literature explicitly tackling this topic of sexual abuse prevention, see Appel, 2023; Grady et al., 2019; Levenson & Grady, 2019; Lievesley & Harper, 2022; Sorrentino & Abramowitz, 2021).

It is not our contention that authors’ personal views should be always explicitly stated, as many researchers come to their topics with a priori assumptions, beliefs, and attitudes. However, it is incumbent on academics to present the reviews of research areas accurately, comprehensively, and in good faith. We do not believe this to be the case when reading Farmer et al.’s work. In conducting their review in such a manner (i.e., with selective reporting, partial and misrepresentative quotation, and self-citation and apparent confirmation bias), the authors of Farmer et al.’s review appear to be promoting a pre-held opinion about the “MAP” literature (and indeed its authors) and only present a perspective that supports this viewpoint.

## Misinterpretation and Misrepresentation

### On Stigma and Risk

We now turn to the most fundamental of our concerns, which relates to the misinterpretation and misrepresentation of academic work related to “MAPs.” The vast majority of Farmer et al.’s review centers the issue of stigma, with this being a common theme across most of this area of research (and in all the papers reviewed by the authors). However, their framing of this concern caricatures the true nature of much of the academic writing on this topic. In perhaps the most flagrant example of this, Farmer et al. state:

For some authors, the stigmatization of sexual interest in children was a primary driver of child sexual abuse. Lievesley and Harper (2022, p. 9) state:

At the core of addressing the social context of prevention is understanding and tackling social stigma about minor attraction. (p. 4084)

There are two key issues to address with this short extract. Firstly, to imply that it is a widely-held view that the stigmatization of attractions to children is a primary driver of child sexual abuse does not represent the level of theoretical knowledge held by those involved in “MAP” research, who predominantly have an academic or professional background in forensic psychology, social work, or clinical psychology (see the above discussion of the motivating/facilitating approach to understand the commission of sexual offenses; Seto, 2019).

Second, the quote offered from Lievesley and Harper (2022) does not actually address the topic of whether stigma is a “primary driver of child sexual abuse”. This extract was actually introducing the idea that the societal context may play an important role in accessing support and abuse prevention services, in the same way that social attitudes can have a facilitating or hindering effect on access to reintegration-related opportunities for people who have committed sexual offenses (for a review of this, see Willis et al., 2010). Lievesley and Harper (2022) applied a well-cited model of desistance from sexual offending that integrates these ideas (see Göbbels et al., 2012) to think about how sexual harm can be prevented before it occurs.

An extrapolation of the argument presented by Farmer et al. is that “MAP” researchers advocate that stigma reduction is a panacea to preventing child sexual abuse. This simultaneously represents both a shallow reading of the literature and a miscommunication of the aims of much of the work conducted on stigma reduction. In an example of this line of argumentation, Farmer et al. state that McKillop and Price’s (2023) argument that dispelling stigma breaks down barriers to support runs “contrary to the long-standing recognition that norms against child sexual abuse have a deterrent and preventative effect” (Farmer et al., 2024, p. 4084). This is a mischaracterization that is clearly evident with just a cursory reading of McKillop and Price’s (2023) writing, which outlines:

. . . stigma also makes it more difficult for MAPs to navigate help-seeking to manage their thoughts and reduce this potential risk. In both instances, opportunities to reduce the risk of CSA [child sexual abuse] perpetration are lost yet are vital to ensure a comprehensive approach to CSA prevention. Part of the solution to these barriers is to educate the community and improve awareness of the importance, and value, of early intervention initiatives that support child safety. Importantly, to do so is not to destigmatize the act of CSA. Rather, it is to enhance community support for implementing upstream interventions to prevent CSA, alongside effective responses when it does occur, to reduce its extent and impact—consistent with a public health prevention model. (p. 705; emphasis added)

And subsequently:

. . . community sentiments and public reactions influence policy professionals and decision-makers, oftentimes leading to a focus on punishment and deterrence after the fact, rather than investment in proactive strategies that seek to prevent CSA from occurring in the first place. This is both short-sighted and potentially harmful by omitting opportunities to intervene early, given CSA is a preventable social problem. The findings in this study raise hope that community education and messaging initiatives may help change misconceptions and attitudes and improve public acceptance of the value of early intervention. . . (pp. 709–710; emphasis added)

Farmer et al., thus, appear to erroneously portray two distinct elements of shame research in the criminological domain as being in competition. The first (and that which is invoked by McKillop & Price, 2023) relates to the need for rehabilitative services to be welcoming and encouraging of service user access. This is a fundamental principle in all healthcare research and has links to issues related to the identification of treatment needs on the one hand, and the importance of a strong therapeutic alliance between a therapist and their client(s) on the other (for a review of the importance of therapeutic alliance, see Baier et al., 2020). The other element of shame research invokes the concept of reintegrative shaming (Braithwaite, 1989), whereby subjective feelings of shame about one’s past offending (or, in the current context, one’s attractions to children) motivate a commitment to non-offending to avoid social and reprisals and adhere to strict social norms (Finkelhor, 2008). It is clear from McKillop and Price’s (2023) paper that they are speaking within the first of these two contexts.

In another example of this, McKillop and Price (2023) outline the importance of considering social responses and stigma toward people with attractions to children in the following way:

In their recent study, Jara and Jeglic (2021) make some important points regarding the need for early intervention and preventative responses to CSA, and the barriers that have impeded access to such support. Drawing on recent studies, they highlight barriers associated with: (1) community misconceptions; (2) stigma; (3) lack of access to support services; and, (4) concerns by MAPs to seek appropriate support. The authors make a compelling argument regarding how these factors, together, create roadblocks to successful implementation of upstream interventions to forestall CSA behavior among those potentially most at risk. (pp. 695–696)

Farmer et al.’s implication that “MAP” researchers argue that most sexually abusive behaviors enacted against children can be attributed in large part to stigma thus sets up an apparent contradiction, where researchers (a) hold that “MAPs” are not at an increased risk to children but (b) are at an increased risk due to stigma associated with their attractions. In actuality, such a contradiction does not exist, and its possibility is only brought about by the straw-manning, misrepresentation, and misinterpretation of the work of those involved in “MAP” research related to stigma.

It is clear from the theoretical literature that sexual attractions can act as a motivator of sexual offending behaviors in people who also have relevant facilitating factors (Seto, 2019), and this is widely acknowledged within the “MAP” literature and the broader forensic psychological evidence base. To imply that “MAP” researchers advocate for such a unidimensional understanding of the drivers of child sexual abuse is, therefore, incredibly misleading. However, it is incontrovertible that anticipated stigma and fears about being reported (which appear to be valid, as evidenced by actual levels of societal hostility and professionals’ and trainees’ stated reporting intentions; see Lievesley et al., 2022; Walker et al., 2021) act as a barrier to people coming forward for support when they otherwise would, for fear of being reported to legal authorities or having their attractions exposed. It is primarily for this reason that anti-stigma interventions are tested in relation to public and professional attitudes toward people with attractions to children (see Harper et al., 2018, 2021; Jara & Jeglic, 2021; McKillop & Price, 2023; for a review, see Lawrence & Willis, 2021).

### On Scientific Accuracy

There are further issues in the reporting of some important findings from the “MAP” literature that Farmer et al. simply get wrong. Fundamentally to their argument, Farmer et al. challenge the extent to which the “MAP” label is favored by communities of people who are attracted to children. They state:

In fact, two surveys of people sexually interested in children have found that they prefer medical terms such as “pedophile” and “hebephile” over the term “minor attraction” (Jahnke et al., 2022; Martijn et al., 2020). (p. 4082).

However, the survey data presented by Jahnke et al. (2022) run contrary to this interpretation, with the “MAP” label being more closely identified with than “pedophile” and “hebephile,” both in terms of self-labeling and the perceived acceptability of labels used by others. In fact, “MAP” was the most strongly endorsed label from a selection of stated options. As such, the statement that people within this community prefer diagnostic labels to be used is incorrect.

When talking about anti-stigma interventions, Farmer et al. state that:

Attempts by MAPs scholars to evaluate interventions designed to destigmatize sexual interest in children and change public attitudes have found that these efforts are ineffective and can result in an increase in negative attitudes (Jara & Jeglic, 2021; McKillop & Price, 2023) (p. 4086).

However, this statement is simply inaccurate. Jara and Jeglic (2021) did find a small but statistically significant difference in attitudes toward “MAPs” after information about them was presented (compared with irrelevant information about substance misuse), where slightly more negative attitudes were associated with the presentation of “MAP” information. However, no baseline measures of attitudes were taken prior to the administration of their experimental manipulation. As such, the implication that such efforts “result in an increase in negative attitudes” confuses correlation with causation, as there is no establishment of whether these participants had more negative attitudes toward “MAPs” to begin with. Addressing this limitation, McKillop and Price (2023) reported a significant improvement in attitudes following a stigma-reduction intervention using a pre–post testing design—the opposite to what is reported by Farmer et al. It is worth noting that a reduction in stigma was also present in McKillop and Price’s (2023) control condition. This may suggest that any observed effects reflect demand characteristics, but the point remains that their data do not reflect a tendency for psychoeducation to increase stigma, as was the argument presented by Farmer et al. Further examples of improvements in attitudes toward people with attractions to children (specifically in the form of pedophilia, which is Farmer et al.’s implied preferred label) come from Harper et al. (2018, 2022), Jahnke et al. (2015), and Lawrence and Willis (2022).

## Conclusions

In this commentary of Farmer et al.’s (2024) critique of the academic use of the “MAP” label in research, we have highlighted several issues that we see as representing significant flaws in their argument. These center primarily around the selective presentation and interpretation of “MAP” scholarship, as well as the misrepresentation of the views expressed by those engaged in this area of work in their writing. As we have shown, the review published in *Trauma, Violence, & Abuse* is both incomplete and misleading and is based on a misunderstanding and superficial reading of the literature. Its publication highlights the importance of both robust and rigorous peer-review processes in this area (e.g., with regard to the appropriate selection of reviewers, and the depth of engagement from reviewers with papers when they are being considered), and of ethical publication practices related to the accurate reporting of others’ work.

As members of the academic community engaged with “MAPs” as our research participants, we hope that this commentary sets the record straight on: (a) the more nuanced and often mundane ways in which “MAP” is actually used by people working in this area and (b) how researchers in this area actually think about central themes related to stigma and risk. To summarize these points, in addition to being a preferred term among people with attractions to children, the “MAP” label is commonly used as an umbrella term in lieu of accurate or suitable diagnostic alternatives, and in a way that reflects the breadth of attractions to children that are observed within this population. On the issue of stigma, “MAP” researchers talk about this as an important treatment need, including how the reduction of stigma can break down barriers to seeking professional support in the pursuit of child abuse prevention. To be clear, “destigmatization” is limited to the attraction, and it is our understanding that researchers are uniformly opposed to the destigmatization and decriminalization of sexual interactions between adults and children. This often places researchers in potential conflict with organizations that would prefer research to not focus on issues related to risk—contrary to Farmer et al.’s assumed complicity between researchers and such organizations.

We also hope that we have identified the importance of rigorous peer-review processes in this area, such that highly contentious claims can be verified prior to the publication of incorrect and misleading information. For example, when writing in such controversial areas, it may be fruitful to speak with authors who are being cited to ensure that interpretations of their work are both accurate and fair. Engaging in such practices would ensure that papers published in high-status journals do not mislead readers who may not be aware of the nuances that exist within such fields.

None of this is to say that contrarian views should not be published. We strongly believe that robust and critical debate is healthy in this area, as this practice enhances research, professional practice, and policy discussions. In writing this commentary, though, we ultimately hope to facilitate a move toward a more transparent, reflective, and (importantly) accurate social science of child sexual abuse prevention.

## Notes

1. We acknowledge that Farmer et al. do include a single sentence citing that “MAP” “could act as a descriptor for any person with a sexual interest in children, a person meeting the diagnostic criteria for pedophilia or a pedophilic disorder, or a person who self-identifies as a MAP.” (p. 4082), before moving on to a detailed discussion that is almost exclusively about destigmatization-related uses of the label.
